# A computational and machine learning approach to identify GPR40-targeting agonists for neurodegenerative disease treatment

**DOI:** 10.1371/journal.pone.0306579

**Published:** 2024-10-08

**Authors:** Arif Jamal Siddiqui, Riadh Badraoui, Mohammed Merae Alshahrani, Mejdi Snoussi, Sadaf Jahan, Maqsood Ahmed Siddiqui, Andleeb Khan, Abdel Moneim E. Sulieman, Mohd Adnan

**Affiliations:** 1 Department of Biology, College of Science, University of Ha’il, Ha’il, Saudi Arabia; 2 Department of Clinical Laboratory Sciences, Faculty of Applied Medical Sciences, Najran University, Najran, Saudi Arabia; 3 Department of Medical Laboratory Sciences, College of Applied Medical Sciences, Majmaah University, Al Majmaah, Saudi Arabia; 4 Department of Zoology, College of Science, King Saud University, Riyadh, Saudi Arabia; 5 Department of Pharmacology and Toxicology, College of Pharmacy, Jazan University, Jazan, Saudi Arabia; 6 Department of Biosciences, Faculty of Science, Integral University, Lucknow, India; Ahram Canadian University, EGYPT

## Abstract

The G protein-coupled receptor 40 (GPR40) is known to exert a significant influence on neurogenesis and neurodevelopment within the central nervous system of both humans and rodents. Research findings indicate that the activation of GPR40 by an agonist has been observed to promote the proliferation and viability of hypothalamus cells in the human body. The objective of the present study is to discover new agonist compounds for the GPR40 protein through the utilization of machine learning and pharmacophore-based screening techniques, in conjunction with other computational methodologies such as docking, molecular dynamics simulations, free energy calculations, and investigations of the free energy landscape. In the course of our investigation, we successfully identified five unreported agonist compounds that exhibit robust docking score, displayed stability in ligand RMSD and consistent hydrogen bonding with the receptor in the MD trajectories. Free energy calculations were observed to be higher than control molecule. The measured binding affinities of compounds namely 1, 3, 4, 6 and 10 were -13.9, -13.5, -13.4, -12.9, and -12.1 Kcal/mol, respectively. The identified molecular agonist that has been found can be assessed in terms of its therapeutic efficacy in the treatment of neurological diseases.

## 1. Introduction

**Fig 1:**
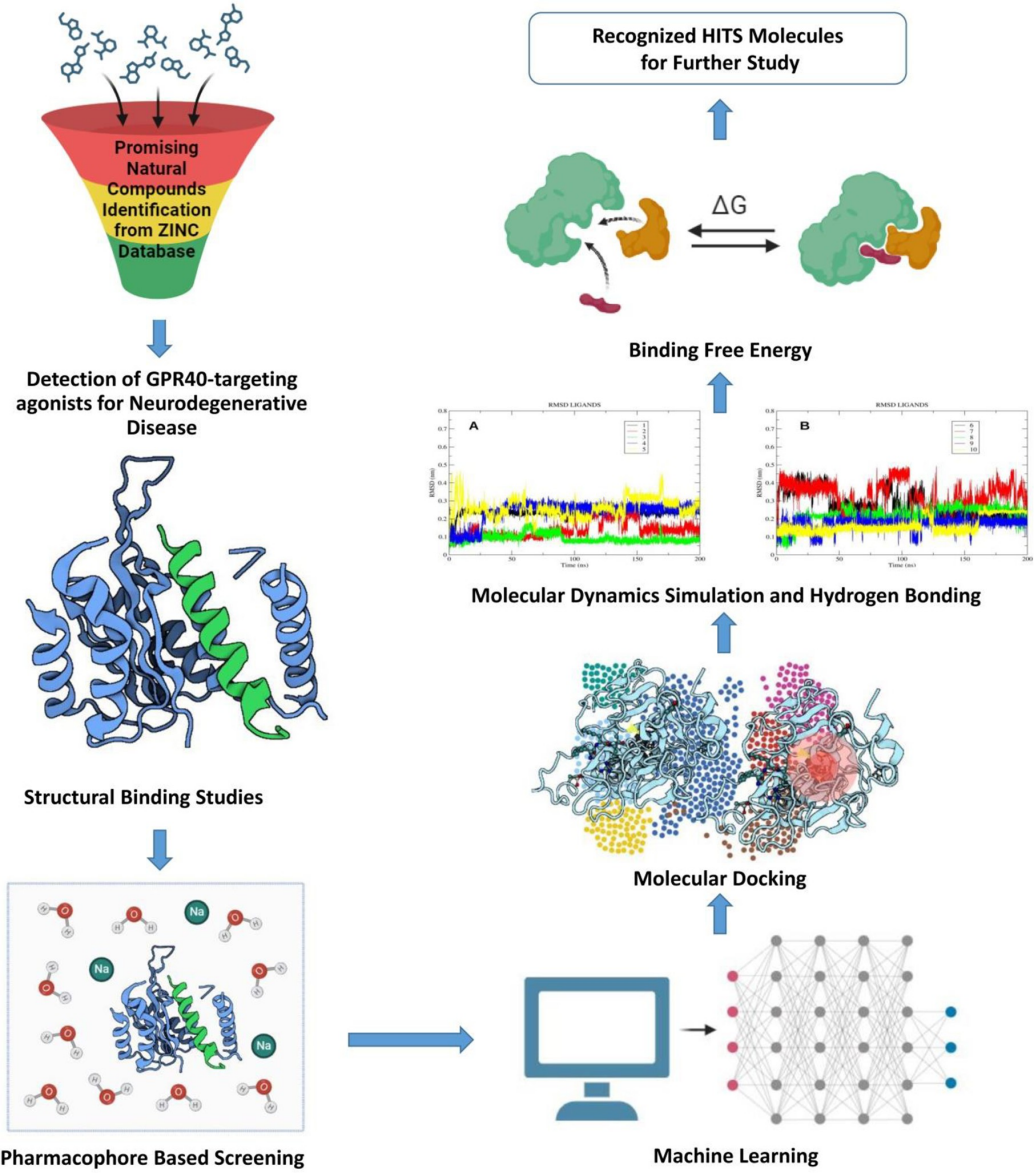
The image indicates the workflow employed for the studies.

Alzheimer’s disease (AD) is a chronic and progressive neurodegenerative disorder characterized by the decline of cognitive abilities and the impairment of memory retention. The conventional pathological indicators of Alzheimer’s disease (AD) consist of extracellular amyloid plaques composed of amyloid-β (Aβ), intracellular neurofibrillary tangles formed by hyper-phosphorylated tau protein, and neuronal loss [1–5]. Research has demonstrated that mutations in the amyloid protein precursor (APP) and presenilin-1/2 (PS 1/2) genes lead to abnormal production of Aβ, which is strongly linked to the development of early onset Alzheimer’s disease (AD) [6, 7]. Furthermore, it has been observed that a decrease in Aβ production resulting from a mutation in the APP gene is associated with a reduced risk of AD in humans [8]. The overwhelming majority of Alzheimer’s disease (AD) cases are considered sporadic in nature and tend to manifest at a significantly later stage in the progression of the disease [9]. There is a pressing need for a novel approach to Alzheimer’s disease (AD) therapy, given that the existing therapeutic choices, such as memantine and cholinesterase inhibitors, solely address the symptoms of the disease without targeting its fundamental causes of progression [10, 11]. The activation of Free Fatty Acid Receptor 1, also known as G Protein-Coupled Receptor 40 (GPR40), occurs primarily in pancreatic beta cells and is triggered by medium- and long-chain free fatty acids [12]. According to the findings of the study conducted by researchers, it was indicated that the activation of GPR40 has the potential to enhance insulin secretion in a glucose-dependent manner [12]. Therefore, GPR40 has emerged as a promising therapeutic target for the treatment of type 2 diabetes. Furthermore, it is worth noting that GPR40 assumes a significant function in the process of neurogenesis and neurodevelopment within the central nervous system of primates and rodents. This includes key regions such as the hypothalamus, cortex, and hippocampus [13]. Kim JY demonstrated that the palmitic acid-bovine serum albumin (PA-BSA) combination has the ability to boost the production of APP and BACE1 by engaging with the GPR40 receptor on SK-N-MC cells and promoting palmitic acid formation [14]. Moreover, previous studies have demonstrated [15] that the activation of GPR40 can enhance the phosphorylation of cyclic adenosine monophosphate response element binding protein (CREB), leading to a significant increase in the expression of neurological factors. Additionally, it has been observed that the GPR40 agonist GW9508 can effectively mitigate cognitive impairments in mouse models of Alzheimer’s disease induced by A1-42 [16]. A more essential discovery is that GPR40 activation may enhance CREB’s degree of phosphorylation, boosting the number of hippocampal neurons in mice [17]. Numerous current research endeavors and investigations have provided substantial evidence regarding the association between GPR40 and Alzheimer’s disease [15, 18–20]. GPR40 is also known to have a significant impact on the process of memory formation and cognitive function [18]. Any modification in the signaling of GPR40 could have substantial ramifications for the processes of learning and memory formation that are impacted by Alzheimer’s disease [19]. There is a substantial body of research indicating that insulin resistance in the brain plays a significant role in the development of Alzheimer’s disease [21–23]. Despite the numerous studies, the precise role of GPR40 in the pathogenesis of Alzheimer’s disease continues to elude researchers. There is still a requirement for the development of effective therapeutic strategies that specifically target GPR40 in the context of Alzheimer’s disease. Currently, individuals diagnosed with Alzheimer’s disease receive treatment aimed at managing cognitive and behavioral symptoms through the use of symptomatic drugs, which are administered with the intention of slowing down the progression of the disease. Despite continuous endeavors to generate more efficacious pharmaceuticals, the significance of GPR40 in the pathophysiology of Alzheimer’s disease renders it a promising candidate for therapeutic discovery and development. Computer-aided drug discovery (CADD) has emerged as a highly effective instrument in the field of drug development, encompassing studies pertaining to Alzheimer’s disease [24–28]. CADD encompasses the utilization of computational methodologies and algorithms to discern possible drug candidates, forecast their interactions with biological targets, and enhance their qualities before doing experimental evaluations. Researchers can expedite the drug discovery process, minimize expenses, and prioritize the most promising drug candidates for experimental validation by utilizing computational methodologies such as target discovery and validation, virtual screening, ADME-Tox prediction, structure-based drug design (SBDD), drug repurposing, and network pharmacology. Therefore, CADD can assume a pivotal position within the drug development process. Computational identification of potential therapeutic candidates, followed by experimental validation, holds promise for the advancement of these candidates to preclinical and clinical trials. This offers a newfound ray of hope for their potential utilization in individuals afflicted with Alzheimer’s disease. The objective of our ongoing research is to ascertain possible agonist compounds that selectively bind to the agonist-binding site of GPR40. The research employs a complex computational methodology that incorporates pharmacophore modeling, virtual screening, molecular docking, molecular dynamics, and a solvation-based scoring scheme. This approach aims to identify prospective compounds that exhibit agonist activity on GPR40, as depicted in Fig 1.

## 2. Materials and methods

### 2.1. Computational structural analysis

Conducting structural investigations is crucial for comprehending the binding pattern and shape of ligands. The conducted experiments facilitate the elucidation of the stability of crucial residues implicated in the interaction between the ligand and the receptor. The structure of the bound ligand with the GPR40 protein was documented in the study, and this information was utilized to identify the crucial binding pocket. In our study, we selected the structures of GPR40 bound to TAK-875 and MK8666 from the RCSB-PDB for our docking simulations. Although TAK-875 and MK8666 are known to function as allosteric agonists, this choice was made based on several key considerations. First, these structures are among the most well-characterized GPR40-ligand complexes available, providing high-resolution data critical for accurate docking simulations. Second, the binding sites of TAK-875 and MK8666 overlap significantly with the orthosteric binding site, offering valuable insights into the receptor’s active conformation and ligand-receptor interactions. Finally, utilizing these structures allows us to leverage their detailed conformational dynamics and binding characteristics, which are essential for identifying potential agonists with similar binding modes. Therefore, despite their allosteric nature, TAK-875 and MK8666-bound structures are suitable and provide a robust framework for our docking studies aimed at discovering new GPR40 agonists. Also, the GP40 PDB utilized was originally associated with type 2 diabetes mellitus research, due to limited availability of GPR40 structures from the brain samples including neurological diseases. The choice of this structure is justified by several factors. Firstly, the fundamental mechanism of GPR40 activation and ligand binding is conserved across different physiological contexts, including metabolic and neurological functions. Thus, the structural insights gained from type 2 diabetes mellitus research remain applicable to neurological studies. Secondly, the selected PDB structures of TAK-875 and MK8666 provide high-resolution data essential for accurate docking simulations, crucial for identifying potential agonists targeting GPR40. Lastly, by leveraging these well-characterized structures, we ensure a robust and reliable framework for our investigation into GPR40’s role in neurological diseases, facilitating the discovery of relevant therapeutic agents despite the initial context of the PDB data. This binding pocket was subsequently employed in computational approaches to facilitate the docking of ligands. Previous research have reported on the examination of structural binding in computational drug design approaches, specifically focusing on the characterization of ligand binding modes within the binding pocket [29–31].

### 2.2. Pharmacophore model generation and validation

The RCSB-PDB database had two inhibitors, specifically TAK-875 (PDBid-4PHU) and MK8666 (PDBid-5TZR), that were reported to bind to the complex of GPR40 [32]. The compounds were employed for the creation of a pharmacophore following a meticulous structural study utilizing molecular dynamics (MD) investigations and trajectory analysis. The Phase module of the Schrodinger software suite was employed to construct pharmacophore hypotheses by analyzing the binding conformations of these drugs. The phase module has the capability to do 3D database searching, structural alignment, and activity prediction. Conformational sampling and a range of scoring methodologies are employed to discern prevalent pharmacophore hypotheses. These hypotheses include the fundamental characteristics of three-dimensional chemical structures that are purportedly crucial for binding, when considering a collection of compounds that exhibit strong affinity towards a certain protein target. Each hypothesis is accompanied with a set of aligned conformations that illustrate the probable method in which the molecules will bind relative to each other.

### 2.3. Virtual screening

The researchers employed a ligand-based pharmacophore model to conduct a screening of the natural product library accessible in the ZINC-15 database [33]. The NP library was obtained and prepared using LigPrep, a ligand preparation feature of the Schrodinger software [34]. The LigPrep methodology encompasses a series of procedures aimed at transforming data, rectifying structures, generating structural variations, eliminating redundant entities, and optimizing molecular configurations. In our present operational process, we employed the Ionizer module to generate ligands while ensuring the preservation of distinct chiralities of the molecules. During the ligand preparation process, high energy ionization states were eliminated. The library that was downloaded consisted of 224205 molecules. Each molecule within the library had up to three distinct conformations, which were determined for each ligand using the OPLS force field [35]. The hits were subsequently subjected to filtration using a structure-assisted ligand-based pharmacophore model. Pharmacophore features were selected based on structural studies and weights were applied to the specific features using the inferences derived from structural studies. Distance constraints were applied to the features along with flexibility to shortlist hit molecules by virtual screening. Only single conformation was shortlisted for each hit molecule.

### 2.4. Machine learning modeling

Activity data for GPR40 protein was downloaded from the CHEMBL database [36]. The downloaded data was processed by removing duplicates and entries having no defined EC50 activities. The "Rdkit" Python utility was utilized for the generation of various descriptors [37]. The detour algorithm is employed for the generation of descriptors, resulting in a reported method that is twice as efficient as alternative approaches. An array of diverse descriptors was computed for the activity data, encompassing descriptors of dimensions 1d, 2d, and 3d, as well as several fingerprint-based descriptors such as pubchem, MACCS, and GraphOnly, for our datasets. A considerable body of literature has been published regarding the utilization of machine learning (ML) techniques in the field of computational drug discovery. The Scikit-learn machine learning package was utilized in this study, employing Python version 3.10 [38, 39]. The dataset was partitioned into training and testing sets using the random shuffle and train test split module provided by scikit-learn. The dataset was partitioned into training and testing sets using an incremental approach, gradually adjusting the train-test ratio from 80/20 to 70/30. This iterative process aimed to maximize the model’s performance by maximizing accuracy. A total of 29 distinct models were assessed in order to determine the optimal model for our dataset based on various statistical criteria. The statistical criteria employed in the analysis included accuracy, receiver operating characteristic (ROC), area under the curve (AUC), and F1 Score for each model. The highest-performing models were employed to evaluate the results acquired from the pharmacophore-based screening of the ZINC database, which was saved as the ZNHT database. The most optimal machine-learning model was employed to conduct a screening of the ZNHT database, which was generated subsequent to the screening of the ZINC database. Following the implementation of this dual screening strategy, we successfully identified a considerable number of compounds that exhibit both a pharmacophore-based profile and a machine learning-based signature pattern. Consequently, the selected hits were further subjected to a more rigorous computational screening procedure based on docking.

### 2.5. Molecular docking

Molecular docking investigations were conducted using the crystal structure of GPR40 in association with the TAK-875 agonist [40]. The docking studies were conducted using the GLIDE docking module of the Schrodinger software suite (version 2022–3) [41]. The Glide software use a search algorithm to identify favorable interactions between ligand molecules and a receptor molecule. These scores provide a quick and efficient means to screen large libraries of compounds, identifying those with the best potential for binding. The Glide system has two distinct docking modes, namely rigid and flexible. The flexible docking mode autonomously generates conformations for any ligand provided as input. The concept of a ligand pose in flexible docking refers to the specific arrangement of a ligand in terms of its position, orientation, and conformation relative to the receptor. The ligand poses generated by Glide undergo evaluation by a series of hierarchical filters that assess the interaction between the ligand and the receptor. The energy-efficient positions are subsequently evaluated and assigned scores at the conclusion. The extra-precision (XP) module of Glide was utilized for the docking process. The XP mode of Glide integrates a rigorous sampling procedure with the utilization of a distinctive scoring function designed to detect ligand poses that are expected to possess unfavorable energies, drawing upon established principles of physical chemistry. The primary objective of the redocking investigations was to optimize the parameters of the docking-based screening methodology. The parameters include “inclusion of input partial charges” into the scoring scheme, “utilization of partial atomic charges”, the method employed for “ligand sampling”, the ability to “sample ring conformations” and “nitrogen inversion” during ligand sampling, the consideration of “intra-molecular hydrogen bonds” as a rewarding factor, the promotion of “planarity in the conjugation of Pi groups”, and the inclusion of “aromatic hydrogen atoms” as potential donors. A grid area with a radius of 10 Å was utilized to build the grid surrounding the ligand. In our investigations, the docking scores were generated using a flexible search strategy in conjunction with XP mode. Several parameters were tuned throughout the docking process in order to obtain a ligand pose that closely overlaps with the binding pose observed in the crystal structure [42, 43]. The Blood Brain Barrier (BBB) penetration prediction of the aforementioned compounds was conducted using the ADMETlab 2.0 website [44].

### 2.6. Similarity index

In order to assess the similarity between the compounds and the ligands already documented in the CHEMBL database [36], we employed the Tanimoto similarity index approach, utilizing the Tanimoto 2d fingerprint [45]. The utilization of 2D fingerprints for similarity searching across different compound activity classes consistently results in a higher number of identified compounds compared to docking calculations. This approach offers computational efficiency and has demonstrated its effectiveness in multiple comparative studies [46]. The Tanimoto coefficient is a widely used metric in the field of molecular similarity analysis, employed to assess the degree of similarity or dissimilarity between molecules. Todeschini et al. conducted a benchmarking analysis wherein they examined 51 similarity coefficients. Their results offer empirical support for the robustness of the Tanimoto coefficient [47]. Python scripts utilizing the RDKIT library were employed to compute the similarity index of the most promising compounds that were chosen following molecular docking investigations [48].

### 2.7. Molecular Dynamics simulation and free energy calculations and ADMET studies

Molecular Dynamics (MD) have the ability to determine the stability of ligands within the binding groove of proteins. This knowledge can be utilized to aid in the discovery of novel ligands that can act as either agonists or inhibitors. MD experiments for ligands [49] were conducted using Gromacs version 2022.4. The GPR40 protein is classified as a transmembrane protein. It is possible to investigate a transmembrane molecule through the application of MD within a conventional solvation model, without the explicit inclusion of the molecule within a membrane protein structure. In MD simulations, the lipid bilayer and the aqueous environment that surrounds a transmembrane molecule are commonly regarded as a cohesive entity with averaged characteristics. This is done by the utilization of a normal solvation model, also referred to as an implicit solvent model or continuum solvent model. When compared to explicit membrane representations, this approach simplifies the simulation setup and has the potential to reduce processing costs. In our studies, the CHARMM-27 force field was employed [50]. Atomic partial charges and position restrain parameters for the ligand were generated using the SWISSPARAM web server. The solvation of the complexes was carried out by placing them in a cubic box with a 1 nanometer radius. The water model used for solvation was an explicit simple-point-charge model. In order to neutralize the system, counterions such as Na+ or Cl^-^ were introduced. The long-range electrostatic interactions were computed with the particle-mesh Ewald method. The evaluation of van der Waals interactions was conducted utilizing the Lennard-Jones 6–12 potential. The bond lengths were constrained using the Linear Constraint Solver approach. In order to resolve the steric clashes between atoms, an extra energy minimization procedure was conducted employing the steepest-descent algorithm for a total of 5,000 iterations. Subsequently, the system was subjected to equilibration utilizing the NVT and NPT ensembles for 200 picosecond respectively, followed by a production run lasting 200 nanoseconds. The trajectory analysis for different complexes was conducted utilizing the built-in facilities in GROMACS, a widely-used molecular dynamics simulation software. Additionally, the XMGRACE tool, available at [https://plasma-gate.weizmann.ac.il/Grace/], was employed to generate graphical representations of the obtained data. The solvation-based scoring of the ligands was performed by utilizing the trajectory of the protein-ligand complex. This was achieved through the application of the widely recognized MMPBSA approach, as described by Valdés-Tresanco et al in their influential publication from 2021 [51]. The software use the AMBER package to conduct the computation of the Gibbs free energy for the protein-ligand complex [51]. The MM-PBSA approach has been found to be more effective in drug discovery compared to conventional free energy estimates [52]. This method takes into account not only the direct interactions between the ligand and the receptor but also solvation effects and entropic contributions, offering a deeper understanding of the stability and energetics of the ligand-receptor complex. The Poisson-Boltzmann equation is commonly employed to approximate the electrostatic properties of biological macromolecules, hence facilitating the investigation of ligand docking score to proteins. The SASA (solvent accessible surface area) approach is employed to determine the region encompassing the protein that is in touch with the solvent sphere through van der Waals interactions. The binding free energy (ΔGbinding) is determined using equations 1, 2, and 3 in this methodology as mentioned in the report [53]. The generation of a free energy landscape was accomplished through the utilization of Python scripts. Initially, covariance was calculated using in built gromacs tool “gmx covar”. The eigenvectors obtained from covariance data were then used to plot the RMS fluctuation per atom on the two principal components which cover more than 95% of the variance in the data. The first 2 principal components are the projections of a trajectory on the eigenvectors of its covariance matrix. gmx sham makes multi-dimensional free-energy plot is generated from these principal components using the gmx sham tool. The gmx sham plots the Gibbs free energy landscapes by inverting multi-dimensional histograms. Further 3d plot was generated using matplotlib library of python. ADMET studies were done using ADMETlab webserver [44].

## 3. Results and discussion

### 3.1. Computational structural analysis

**Fig 2:**
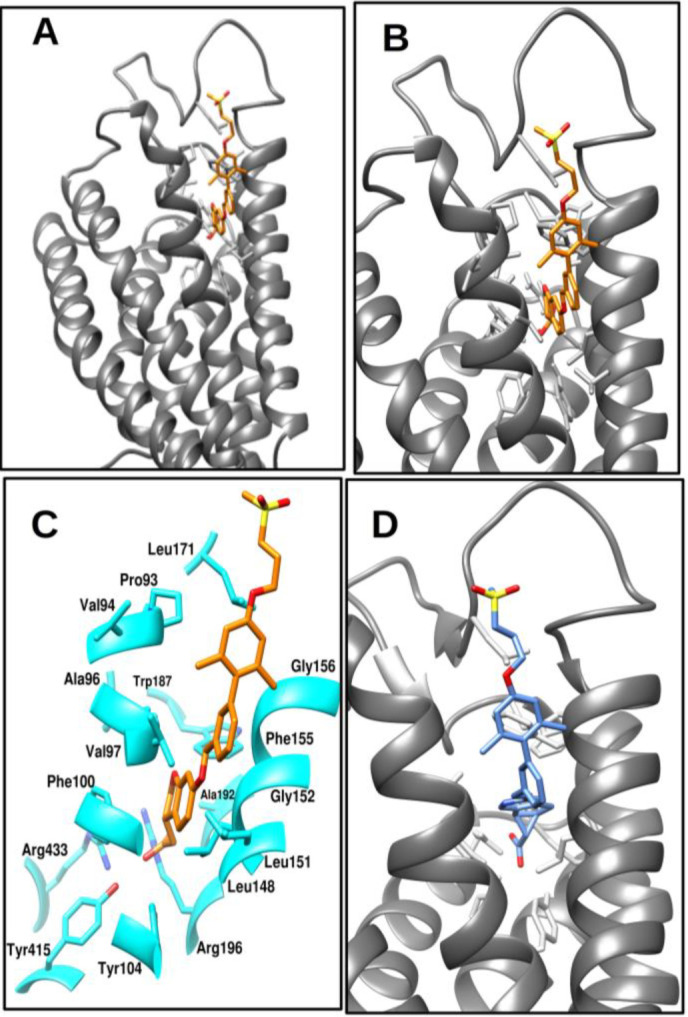
The image A indicates the ribbon view of protein and agonist binding bound cavity with bound ligand TAK-875. **A-D.** Image B indicate the enlarged view of cavity, while the image C indicates the residues observed to be interacting with ligand (orange color). Image D indicates the bound structure of MK-8666.

A limited number of agonist ligand compounds, specifically two, have been described for the GPR40 protein. One compound, TAK-875, had a crystal resolution of 2.332 Å as determined by the Protein Data Bank (PDB) entry 4PHU. The other compound, MK-8666, displayed a resolution of 2.2 Å according to PDB entry 5TZR. Both ligands were shown to interact with the identical allosteric binding site on GPR40, as depicted in Fig 2A–2D. The protein TAK-875 was targeted by agonists, which successfully progressed to phase II and phase III of clinical trials. However, the agonist was ultimately removed during phase III due to the occurrence of drug-induced liver injury. In comparison, MK-8666 had agonistic properties towards GPR40; however, it was subsequently discontinued during the phase I clinical trials. Additionally, following alignment, both structures exhibited a significantly low root mean square deviation (RMSD) value of 0.260 Å. The ligand-bound conformation of GPR40 with TAK-875 was chosen for our research, with TAK-875 serving as the control molecule. The PDB entry with the identifier 4PHU has a gap in the amino acid sequence, namely from residue 111 to residue 120. The residues were modeled with the SwissModel web server [54].

The model underwent energy minimization in order to eliminate undesirable interactions, utilizing the "Protein preparation wizard" tool within the Schrodinger software. Extended molecular dynamics (MD) simulations were conducted for a duration of 1 microsecond in order to meticulously examine the time dependent stability of interaction between the protein and ligand, as well as identify the crucial residues involved in the receptor contact. The interaction between the agonist and specific residues, including Pro80, Val81, Ala82, Phe87, Tyr91, Leu135, Leu138, Gly139, Phe142, Gly143, Leu168, Trp174, Ala179, Arg183, Tyr2240, and Arg2258, was detected.

**Fig 3:**
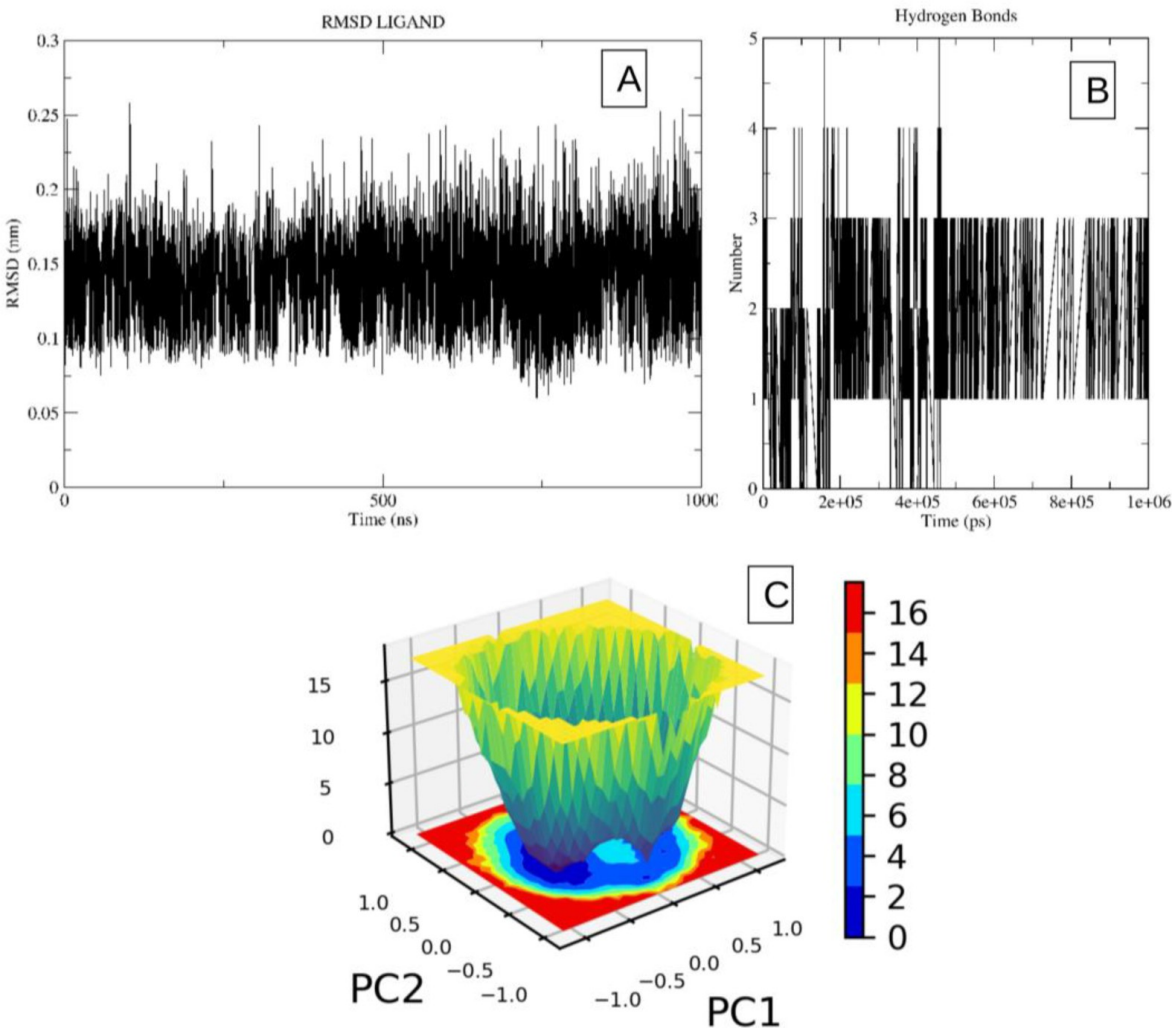
The image 3A indicates the RMSD plot of molecule TAK-875 in bound conformation with the receptor, while the image 3B indicates the hydrogen bond pattern observed during the simulation time frame. A-C. Image 3C indicates the free energy landscape plot of the ligand.

The stability of the system was determined through the analysis of its root mean square deviation (RMSD), which exhibited a low value of 2.1 Å (Fig 3A). The stability of both the protein receptor and the ligand conformations was found to be significant. Furthermore, the molecule was often observed to form around 1–3 hydrogen bonds with the receptor during the simulation, as depicted in Fig 3B. It is important to acknowledge that compounds that bind to the agonist binding site of this receptor have the potential to boost downstream signaling mediated by the GPR40 molecule, similar to the effects observed with TAK-875 and MK-8666. The plot of the free energy landscape also demonstrates the presence of a single global minimum for the molecule closely followed by another low energy conformation. Although, there was minor change in conformation between the two conformations indicating high stability (Fig 3C). Consequently, the molecular dynamics (MD) investigations have indicated the potential for conducting additional computational studies, such as docking and pharmacological modeling, on the binding site of the protein. Furthermore, the conformation of the agonist can be utilized to construct a ligand-based pharmacophore by incorporating specific interactions observed during simulation studies.

### 3.2. Pharmacophore model generation and validation

**Fig 4:**
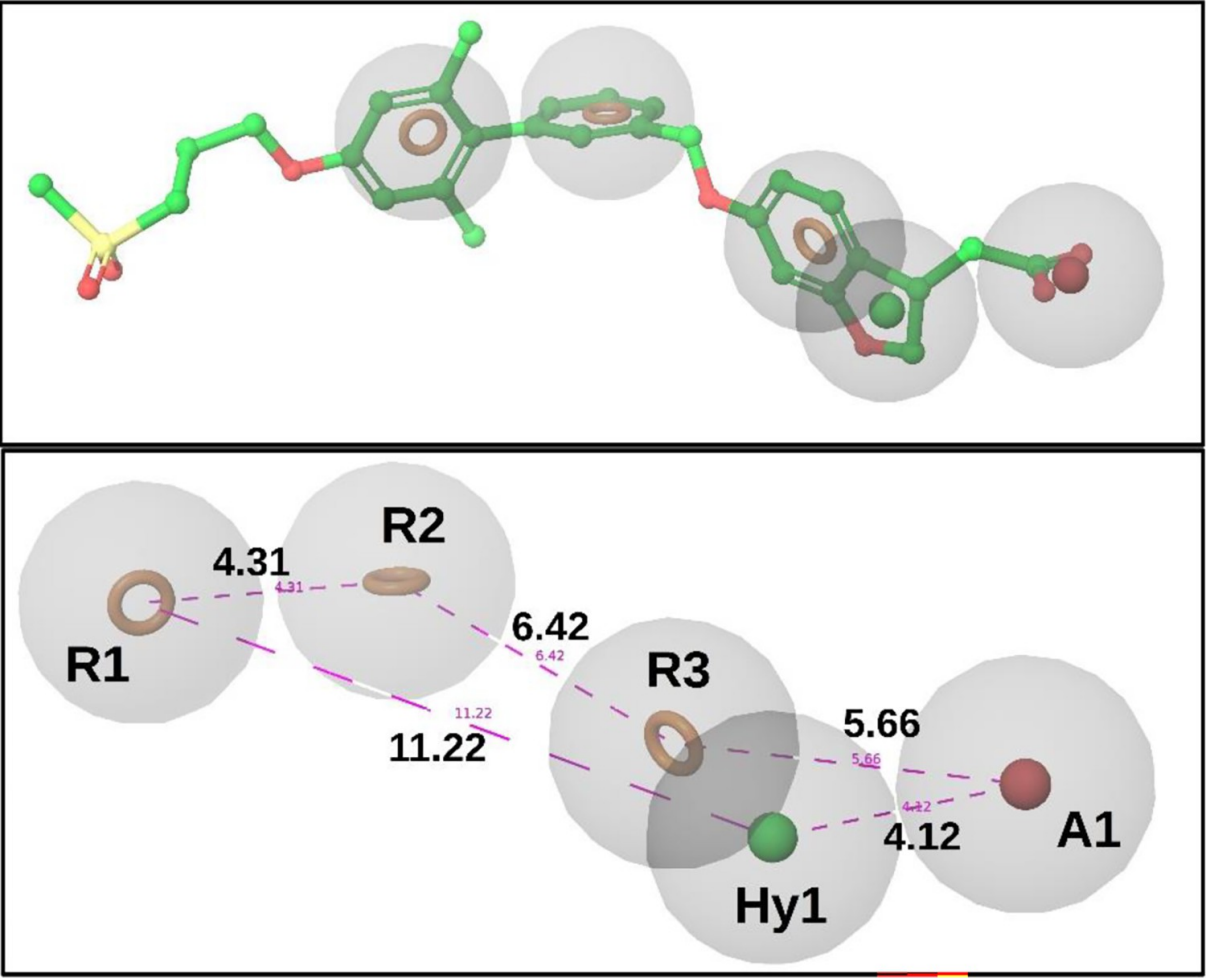
The image indicates the pharmacophore model developed by using extensive structural ligand studies.

The crystal structures of the TAK-875 and MK-8666 bound receptor complexes were utilized in order to gain insight into the crucial residue interactions that contribute to the stability of the agonist within the binding groove of the GPR40 protein. The contact between the carboxylic group and the Tyr2240 and Arg183 residues was detected, suggesting that the carboxylic group could serve as an Acceptor group to establish crucial interactions with these residues. In a similar vein, it has been noted that the hydrophobic portion of TAK-875, when situated near the hydrophobic groove of the protein, can serve as a hydrophobic characteristic. Furthermore, the strategic positioning of an aromatic group in close proximity to a hydrophobic feature can potentially enhance the preservation of pi-pi interactions with Tyr91 and Phe87. Consequently, an aromatic characteristic was incorporated into the pharmacophore model. Two additional aromatic features were incorporated into the model in order to maintain interactions with Phe142 and Trp174. By integrating the aforementioned features and incorporating distance constraints, we have developed a pharmacophore model consisting of five features. This model will be utilized in our subsequent computational screening methodology. Distance constraints were implemented on the pharmacophore model, accompanied by a tolerance range of 10–15% for certain attributes. This was done to introduce flexibility in the search criteria throughout the process of screening the database. The pharmacophore model consisted of three aromatic features (R1, R2, and R3), one hydrophobic feature (Hy1), and one acceptor feature (A1) (see Fig 4). Three distinct variations of the pharmacophore model were developed by altering the tolerance and constraints parameters. Among the three models under consideration, it is seen that model 2 exhibited the most elevated statistical values, as indicated in Table 1. The employed model for screening purposes was a validated structure-guided approach.

**Table 1:** This table summarizes the statistical parameters for pharmacophore models of GPR40 test database containing active and decoy sets.

Parameters	Model-1	Model-2	Model-3
Total no. of molecule s in database (D)	100	100	100
Total no. of active (A)	20	20	20
Total hits (H_t_)	28	24	30
Active hits (H_a_)	17	18	16
% yield of actives (H _a_ / H _t_ × 100)	60.09	75	53.33
% ratio of actives (H _a_ / A × 100)	85	90	80
Enrichment factor (EF)	3.03	3.75	2.66
False positives (H_t_—H_a_)	9	6	14
False negatives (A—H_a_)	3	2	4
Goodness of hit score (GH)	0.59	0.73	0.50

### 3.3. Virtual screening

In the virtual screening technique, it was deemed necessary to evaluate four specific features, whereas one feature was deemed optional for the evaluation of molecular pharmacophore features. By applying the criteria, we successfully filtered out 3772 results from a total of over 0.20 million compounds in the natural product database. Given that the proportion of finds accounted for a mere 0.0187% of the total search area, we proceeded to expose all identified hits to a more rigorous screening approach. This involved evaluating the hits through the utilization of the extra precision (XP) docking methodology of glide. The excluded search results were converted into sdf file format and subsequently transformed into a ZNHT database using the LigPrep module. The database underwent additional machine learning-based filtering, as detailed in the section below.

### 3.4. Machine learning modelling

**Fig 5:**
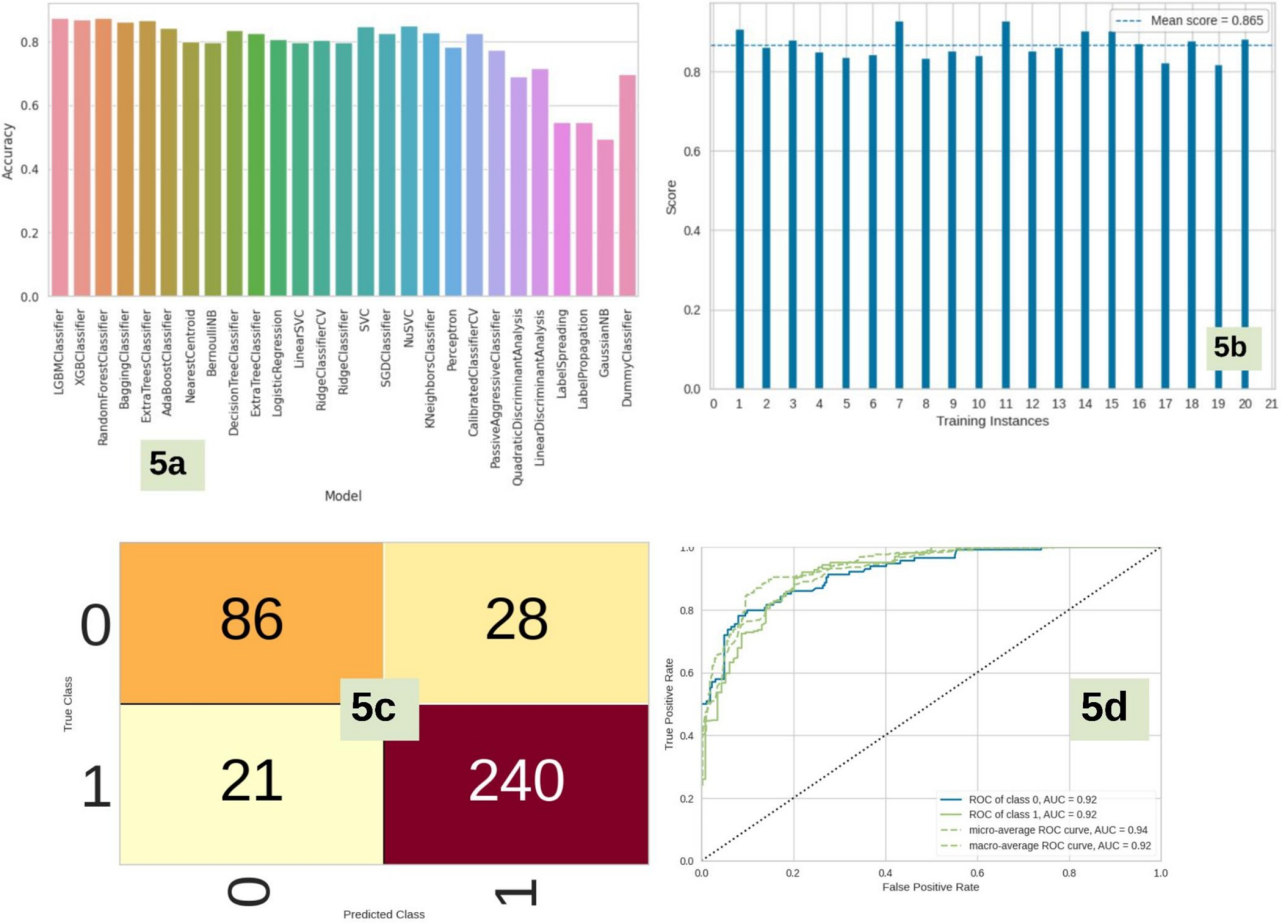
**a-d.** The image “5a” indicates the accuracy plot of various ML models represented in the form of vertical bar plot. Image “5b” indicates the cross-validation score of “LGBMC Classifier” model. Image “5c” indicates the confusion matrix of “LGBMC Classifier” model, which displayed the best accuracy. Image “5d” indicates the ROC curve of “LGBMC Classifier” model.

CHEMBL id–CHEMBL4422 was used to download the activity data of GPR40 molecules. The entire dataset is partitioned into training and test sets, with a gradual adjustment of the ratio from 80/20 to 70/30, in order to improve the model and achieve the highest level of accuracy. In our binary classification approach, compounds with an EC50 value of 1 μM or less were categorized as active, whereas molecules with an EC50 value above 1 μM were categorized as inactive. The dataset was utilized to construct a binary classification model employing a selection of prominent machine learning methods such as random forest, support vector machine, ada boost classifier, extra tree classifier, decision tree classifier, bagging classifier, and others. Out of the 29 distinct algorithms examined, LGBMC exhibited the highest levels of accuracy (0.87), ROC (0.92), AUC (0.92), and F1 score (0.87). The XGBoost classifier and random forest classifier were closely observed, as depicted in Fig 5A. The LGBMC classifier demonstrated a significantly high cross-validation score of 0.865 when evaluated using a 20-fold cross-validation technique (Fig 5B). The LGBMC classifier model that was chosen as the best performer had a notable level of accuracy in both training and test phases, as seen by the confusion matrix (Fig 5C). The model demonstrated a high level of precision in accurately identifying inactive chemicals (0.804), with a slightly higher precision observed in the identification of active molecules (0.896). The active compounds exhibited a memory value of 0.920, while the inactive molecules had a slightly lower recall value of 0.804. The area under the receiver operating characteristic (ROC) curve was determined to be 0.92 for both the active and inactive classes, as depicted in Fig 5D. The LGBMC Classifier model was chosen for binary classification of the ZNHT database, which was constructed from the virtual screening of the ZINC database using a pharmacophore-based technique, based on the statistical characteristics. Following the utilization of machine learning techniques for virtual screening of compounds, a total of 2432 active molecules were obtained.

These molecules had similarities in their descriptor-based data to molecules that have been previously reported in the CHEMBL database, with an EC_50_ value below 1 μM. The database was effectively decreased to 64.47% of its initial size. The compounds were preserved in the ZNHT_2 database for subsequent computational investigations.

### 3.5. Molecular docking

The crystal structure of GPR40 complexed with the TAK-875 agonist, identified by the Protein Data Bank (PDB) identifier 4PHU, was employed in our investigation of structural binding. This crystal structure served as the basis for conducting docking investigations. The Glide XP docking approach was employed to assess the docking score of compounds to the receptor through docking. Various parameters were tuned in the glide tool to achieve the most optimal redocking stance. The input partial charges were included into the scoring scheme, while partial atomic charges were not utilized. Ligand sampling was done by including sample ring conformations only while the “nitrogen inversion” were not included. Intra-molecular hydrogen bonds were not rewarded in scoring scheme and conjugate-Pi groups were made planar. Also, the “aromatic hydrogen atoms” were selected as potential donors during redocking studies.

**Fig 6:**
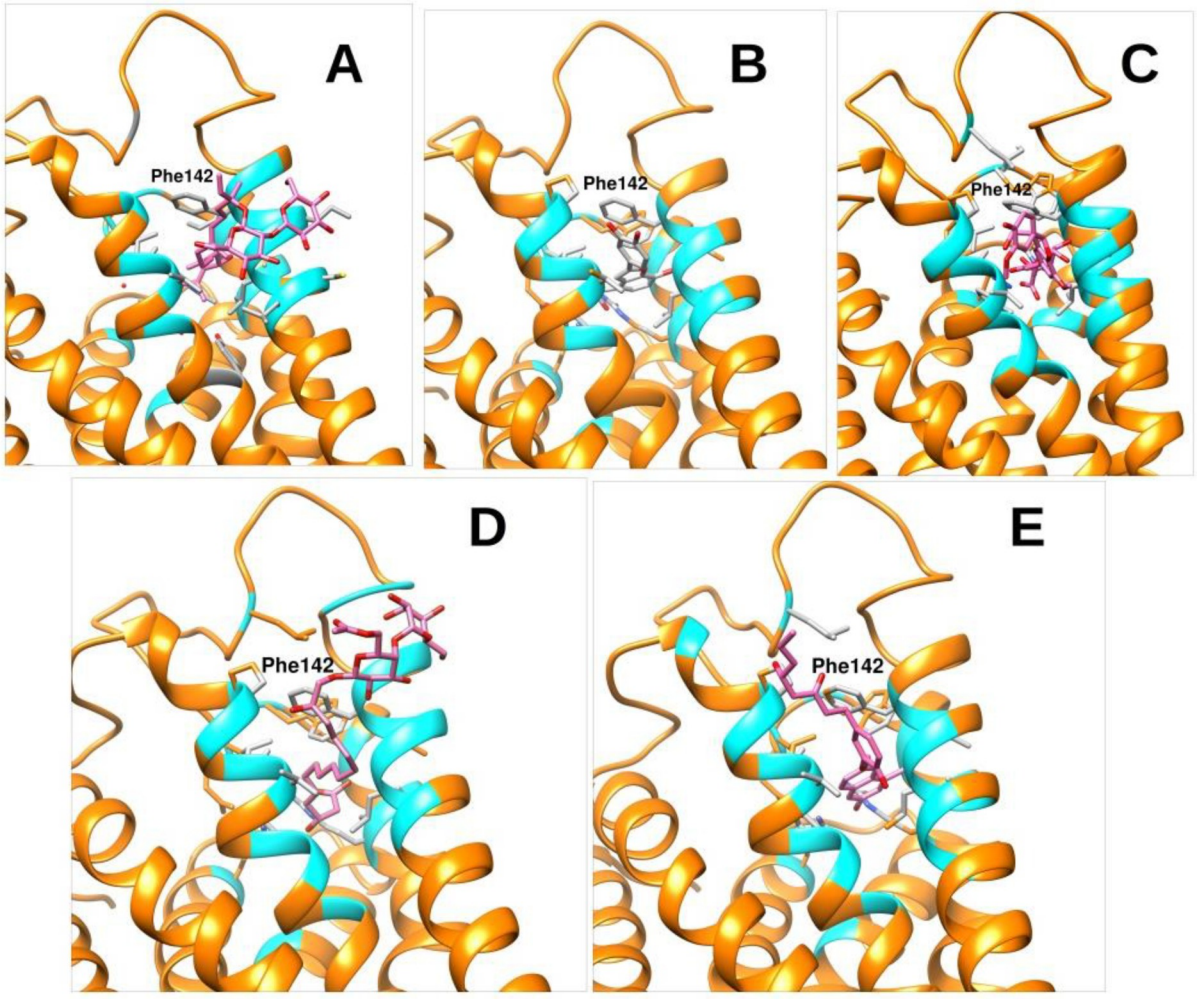
The image 6A – 6E indicates the binding poses of the top selected molecules namely 1, 3, 4, 6 and 10 respectively. A-E.

Upon meticulous optimization of the docking parameters, we successfully generated the docking pose exhibiting a root-mean-square deviation (RMSD) of less than 0.14 Å. The redocking stance exhibited a docking score of -12.1 Kcal/mol. Following the process of docking-based screening, it was revealed that a mere 10 molecules exhibited a superior docking score compared to TAK-875. The compounds were subjected to meticulous analysis to assess their potential for molecular interaction with the receptor, as depicted in Fig 6A–6E. The observation was made that all compounds exhibited binding to the identical binding pocket of the GPR40 protein, albeit with diverse binding conformations. The compound with the highest selection score displayed a docking score of -13.9 Kcal/mol, while molecule 2 exhibited a docking score of -13.8 Kcal/mol. Compound 1 was observed to engage in hydrogen bonding interactions with the backbone of residue Gly139, as well as with the side chains of residues Arg183 and Arg420. It also displayed hydrophobic Interactions with residues namely Ala96 (3.9 Å), Val97 (4.2 Å), Leu151 (4.5 Å), Leu184 (4.6 Å), Phe100 (4.4 Å), Phe155 (4.7 Å). Compound 3 exhibited hydrogen bonding interactions with the side chains of Arg183 and Arg2258, as well as the backbone atoms of Pro80 and Leu138. Compound 4 exhibited the ability to engage in hydrogen bonding interactions with the side chains of Tyr91, Tyr2240, and Arg183, as well as with the backbone of Pro93. Compound 6 exhibited interactions with certain residues in the protein backbone, including Pro80, Gly139, Leu140, and Gly143, as well as with the side chains of Ty91, Arg183, and Arg2258. Compound 10 was observed to engage in hydrogen bonding interactions with both the side chain of Arg183 and the backbone of Pro80. Additionally, Pi-Pi stacking interactions were reported with residue Phe87. The binding affinities and the details of the hydrogen and hydrophobic interactions of the top selected compounds have been compiled and shown in Table 2. In summary, Hydrogen bond interactions feature prominently with Arg183, often forming bonds with distances between 1.7 and 2.5 Å and angles ranging from 99 to 148 degrees. Other significant hydrogen bonding residues include Tyr91, Arg2258, and Tyr2240, demonstrating varying bond angles and distances. Residues such as Gly139 and Glu172 also appear frequently, indicating their role in stabilizing ligand binding through hydrogen bonds. Hydrophobic interactions were predominantly observed with residues Ala83, Val84, Leu138, Phe142, and Leu171, all showing frequent participation with distances ranging from 3.2 to 4.9 Å. Notably, Phe87, Trp150, and Val141 also contribute significantly. The prediction of the Blood Brain Barrier (BBB) for the compounds was conducted using the ADMETlab 2.0 website. All the compounds had excellent blood-brain barrier (BBB) penetration values and demonstrated similar results to the TAK-875 inhibitor, as shown in Table 3. The presented compounds exhibited similar or favorable blood-brain barrier (BBB) penetration values across all molecules. The molecules underwent molecular dynamics (MD) experiments, as outlined in the subsequent section.

**Table 2:** Table 2 indicates the binding affinities as well as MMPBSA score of top selected molecules along with the summary of residues involved in the Hydrogen and hydrophobic interactions. Distance was measured in Armstrong and angle was measured in degrees (°) from donor to acceptor atoms for hydrogen bonds.

Sr. No.	Code Used	ZINC Code	Glide Docking Score	MMPBSA Score	Hydrogen Bond (Distance, Angle)	Hydrophobic Interactions (Distance)
1	1	ZINC000067913631	-13.9	-63.3	Arg183(1.7, 148); Arg2258(2.6, 124); Gly139(2.3, 104)	Ala83(3.9); Val84(4.2); Leu138(4.5); Leu171(4.6); Phe87(4.4); Phe142(4.7)
2	2	ZINC000038238855	-13.9	-40.3	Arg183(1.9, 135); Glu172(2.6, 148)	Ala83(3.8); Leu138(4.6); Leu171(4.7); Phe142(4.5)
3	3	ZINC000095919068	-13.5	-47.6	Arg183(2.0, 116); Glu172(1.8, 129); Arg2258(2.6, 130); Pro80(2.3, 115); Leu138(1.9, 104)	Ala83(4.5); Val84(4.3); Phe142(2.9); Leu138(4.2);
4	4	ZINC000067910850	-13.4	-59.9	Tyr91(2.8, 120); Arg183(2.5, 120); Tyr2240(2.2, 124); Ala189(2.5, 106); Leu135(2.6, 114); Pro80(2.6, 100)	Phe142(4.2); Gly139(4.3); Val84(4.1)
5	5	ZINC000604377487	-13.4	-44.2	Tyr91(2.3, 107); Arg183(1.7, 105); Arg2258(2.1, 145): Gly148(2.1, 135);	Ala83(4.2); Phe87(2.8); Leu138(4.4);Phe142(4.3)
6	6	ZINC000604377474	-12.9	-54.8	Arg183(1.7, 133); Tyr2240(2.7, 111); Gly139(3.0, 148)	Ala83(4.2); Trp150(3.2); Phe142(4.7)
7	7	ZINC000604377556	-12.7	-38.2	Tyr91(2.2, 120); Arg183(2.0, 109); Tyr2240(2.7, 198); Arg2258(1.9, 96)	Ala83(4.0); Val84(4.8); Leu138(3.9)
8	8	ZINC000085625919	-12.3	-27.5	Arg183(1.8, 122); Ala83(1.9, 99); Gly139(2.7, 108); Ala179(2.7, 110)	Phe87(4.7); Val141(4.4); Ala182(4.0); Val84(4.7); Leu138(4.9); Trp174(4.6); Leu171(4.7)
9	9	ZINC000085597495	-12.1	-28.8	Arg196(2.3, 113); Ala192(2.5, 109)	Ala83(4.4); Val84(3.7); Leu138(4.2)
10	10	ZINC000085597488	-12.1	-45.2	Arg196(1.9, 112); Leu151(2.6, 104)	Phe142(4.4); Phe87(4.4); Leu138(4.4); Ala182(4.3)
11	Control	TAK-875	-12.1	-42.5		

**Table 3:** The table summarizes the ADMET values of various top shortlisted hit molecules after docking based evaluation.

Sr. No.	ZINC DATABASE CODE	Code used	logP	F30%	BBB Penetration	H-HT	T 1/2
1	ZINC67913631	1	0.059	0.999	0.349	0.172	0.882
2	ZINC000038238855	2	-1.894	0.999	0.16	0.277	0.951
3	ZINC000095919068	3	3.695	1.0	0.008	0.613	0.915
4	ZINC000067910850	4	0.884	0.993	0.278	0.733	0.751
5	ZINC000604377487	5	-0.165	1.0	0.246	0.098	0.841
6	ZINC000604377474	6	-0.146	1.0	0.264	0.134	0.888
7	ZINC000604377556	7	0.16	1.0	0.252	0.081	0.869
8	ZINC000085625919	8	2.37	0.961	0.04	0.38	0.907
9	ZINC000085597495	9	2.608	0.995	0.049	0.597	0.863
10	ZINC000085597488	10	4.646	0.785	0.058	0.586	0.644
11	**Control**	Control	2.286	0.878	0.873	0.986	0.848

### 3.6. Similarity index calculations

The RDKit module in Python was utilized to compute the chemical similarity index between a set of known inhibitors described in CHEMBL that exhibit agonist action against the GPR40 protein. It was observed that all compounds had a similarity index < 71% when compared to the reported molecules, as measured by both the Tanimoto MACCS value and the Tanimoto MORGAN value. Compounds that possess structural similarities typically exhibit a tendency to interact with proteins that share comparable characteristics. Research conducted by Martin et al. has demonstrated that there exists a 30% likelihood that a molecule exhibiting a resemblance of ≥0.85 to a previously reported compound will exhibit activity [55]. Therefore, all the compounds possess unique structures and have not been previously documented as having activity against the GPR40 protein. The molecular similarity data has been compiled and shown in Table 4.

**Table 4:** The table summarizes the Tanimoto similarity index of all selected top molecules.

Sr. No.	Compounds Name/code	Tanimoto-MACCS	Tanimoto-MORGAN
1	ZINC000067913631	0.479452	0.128205
2	ZINC000038238855	0.586957	0.170732
3	ZINC000095919068	0.555556	0.284211
4	ZINC000067910850	0.559322	0.270968
5	ZINC000604377487	0.5	0.284615
6	ZINC000604377474	0.666667	0.443114
7	ZINC000604377556	0.487805	0.285714
8	ZINC000085625919	0.652174	0.296875
9	ZINC000085597495	0.675	0.383333
10	ZINC000085597488	0.707317	0.37963
11	TAK-875	Control	Control

### 3.7. Molecular Dynamics simulation and free energy studies

**Fig 7:**
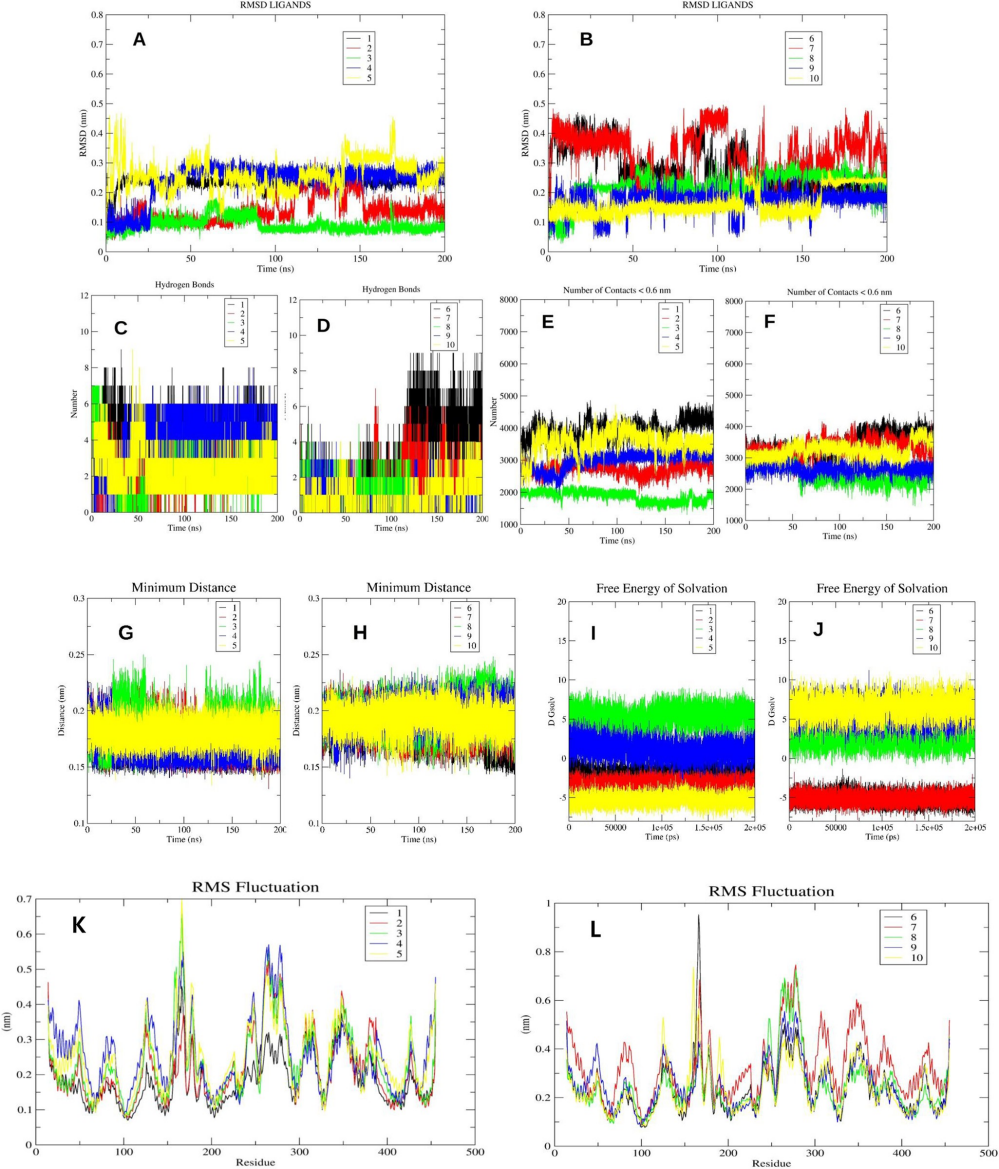
**A-L.** The image 7A and 7B indicates the RMSD plots of the ligands conformations when bound to receptor. The image 7C and 7D indicates the hydrogen bond plots of the molecules. Image 7E and 7F indicate the total number of contacts observed between the protein and the ligands during simulation. Image 7G and 7H indicate the minimum distance observed between the protein and the ligands during MD. Image 7I and 7J indicate the free energy of solvation of the ligand molecules observed during MD studies. Image 7K and 7L indicate the RMSF of the residues in the presence of the ligands.

Additional compounds that were selected as potential candidates underwent molecular dynamics (MD) simulations using the Gromacs 2022.4 software package. Upon conducting a meticulous analysis of the trajectories of various molecules, it was observed that certain molecules, which were included in the final selection, exhibited stable root mean square deviation (RMSD) plots with RMSD values below 3 Armstrong (as depicted in Fig 7A). The ligands, specifically 1, 3, 4, 8, and 9, demonstrated stable root mean square deviation (RMSD) plots throughout the simulation. Minor conformational changes were observed, with RMSD values below 3 Å, demonstrating the ligands’ significant stability in their poses during the simulation (Fig 7B). During the course of a 200 ns molecular dynamics (MD) simulation, compounds 2, 6, and 10 were observed to adopt stable conformations. Compounds 5 and 7 exhibited a lack of stability in achieving a stable conformation during the molecular dynamics (MD) simulation, suggesting a low level of pose stability. During the simulation investigations, the chemicals designated as 1, 3, 4, 8, and 10 were observed to establish 5, 4, 6, 3, and 3 hydrogen bonds, respectively, with the receptor, as depicted in Fig 7C–7D. It is noteworthy that compound 6, upon attaining a stable conformation at around 120 nanoseconds, established a maximum of eight hydrogen bonds with the receptor during the duration of the simulated investigations. During the molecular dynamics (MD) simulation, it was observed that the formation of hydrogen bonds in molecules 2 and 9 was rather infrequent. The ligand-receptor complex typically maintains a minimum distance of less than 2.5 Å, suggesting a high likelihood of non-covalent interactions. This is consistent with the general observation that non-covalent interactions predominantly occur within 4 Å, as depicted in Fig 7E–7H. Additionally, the measured number of connections approached approximately 4000 for molecules 1, 4, 5, 6, 7, and 10, suggesting the presence of extremely favorable interactions among these molecules. Furthermore, it was noted that there were no significant changes in the free energy of solvation for all the ligands that were bound (Fig 7I, 7J). This suggests that there were no substantial alterations in the binding orientation of the molecules throughout the simulations. Also, mostly protein residues involved in binding displayed similar root mean square fluctuations in presence of different residues except for ligand 7 (Fig 7K, 7L). During our observation on molecular trajectories, it was seen that molecules 1, 3, and 6 exhibited stable hydrogen bond interactions with both Arg183 and Arg2258. Compound 4 and 10 were found to exhibit stable hydrogen bonds with Arg183, as reported previously [56, 57].

**Fig 8:**
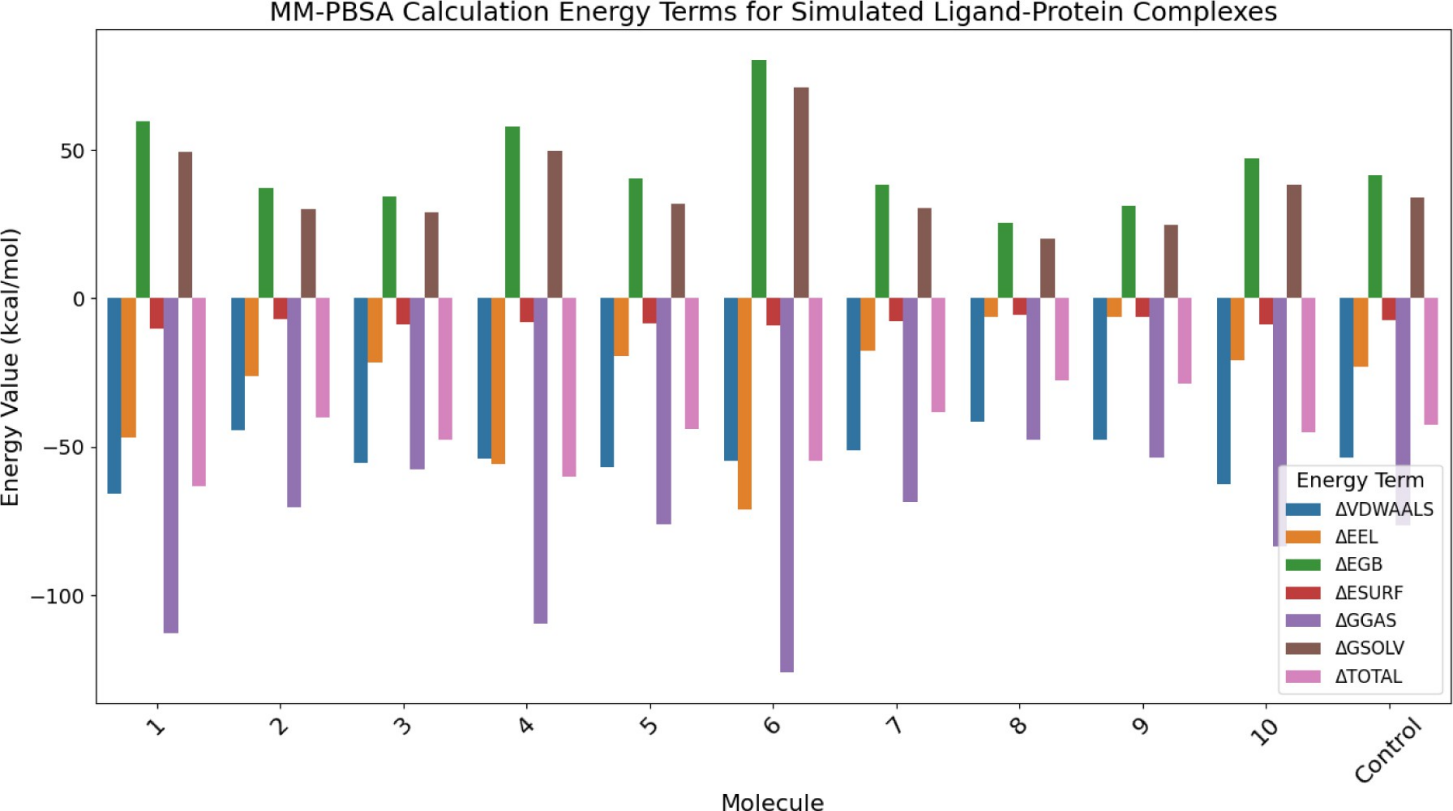
The image summarizes the MM-PBSA calculation energy terms for various ligands.

**Fig 9:**
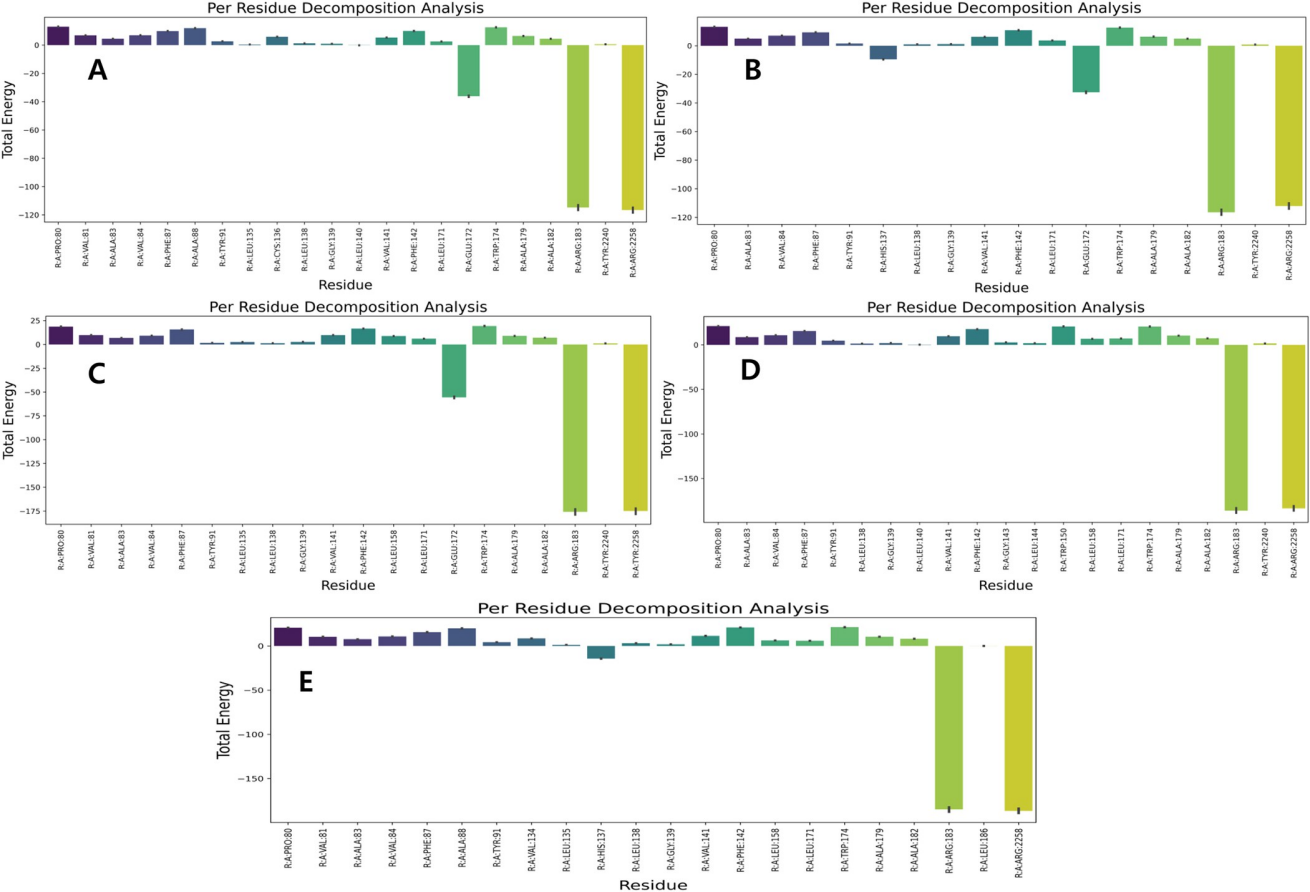
A-E. The image 9A to 9E indicates the per residue decomposition analysis of the compounds namely 1, 3, 4, 6, 10.

**Fig 10:**
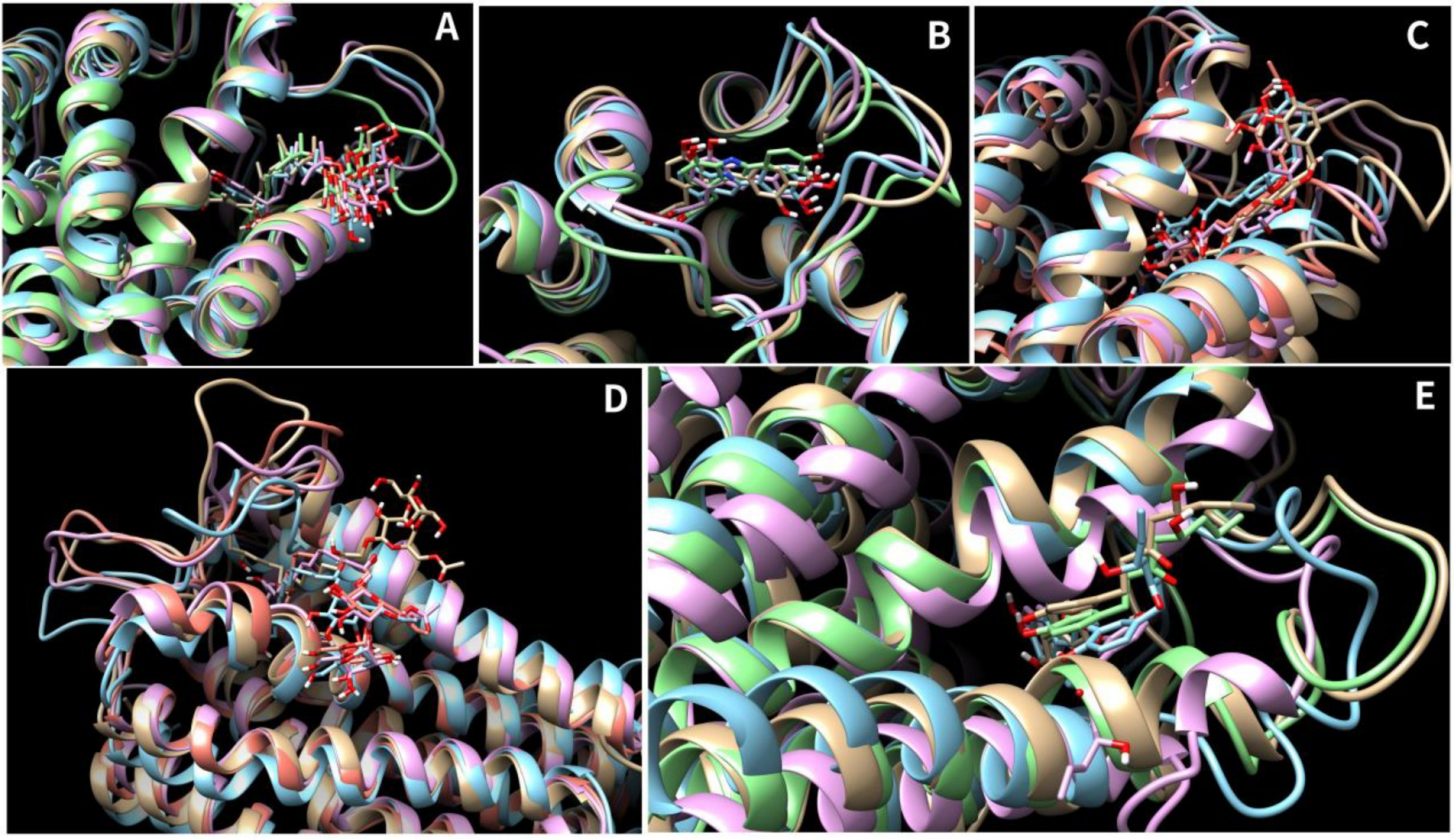
**A-E**. The image 10A to 10E indicates the overlapped poses of the 50ns, 100ns, 150ns and 200ns time frames of the compounds namely 1, 3, 4, 6, 10.

**Fig 11:**
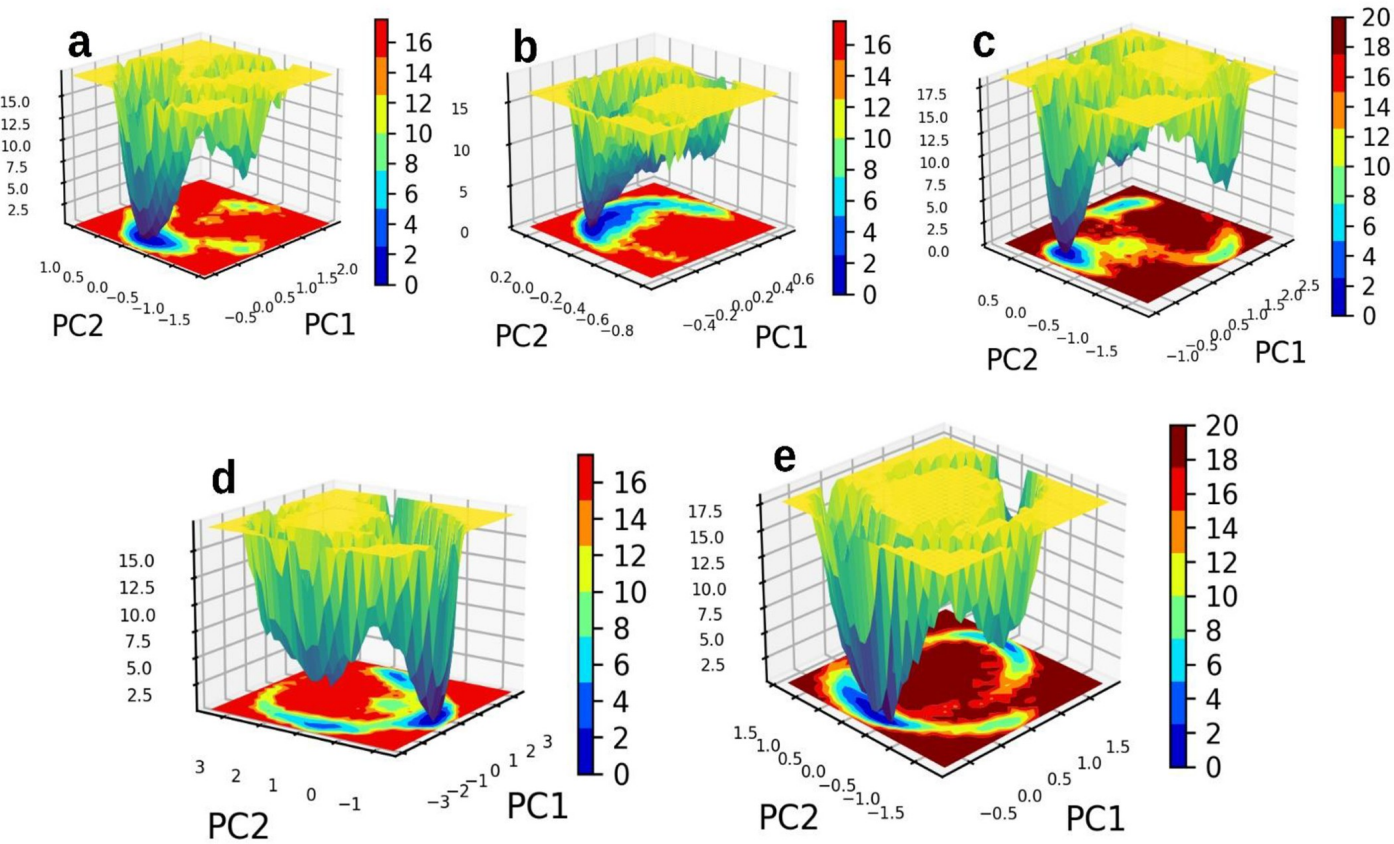
**a-e.** The image 11a to 11e indicates the free energy landscape plots of the compounds namely 1, 3, 4, 6, 10 when bound to receptor.

The results of the MMPBSA calculations revealed that six compounds had a greater solvation based binding affinity compared to the control molecule TAK-875. The compounds, specifically molecules 1, 3, 4, 5, 6, and 10, exhibited solvation-based binding affinities with values of -63.3, -47.3, -59.9, -44.2, -54.8, and -45.2, respectively. The complete details of various energetic components have been summarized in Fig 8 and Table 4. In the analysis of the molecular dynamics (MD) simulations, our focus was on evaluating the stability and interaction patterns of the ligand-receptor complexes. We observed stable root mean square deviation (RMSD) plots for several compounds, indicative of their robust binding to the receptor. Notably, compounds 1, 3, 4, 8, and 9 displayed consistent RMSD values below 3 Å, suggesting minimal conformational changes and significant stability in their poses throughout the simulation period (Fig 7A). Similarly, compounds 2, 6, and 10 demonstrated stable conformations, albeit with minor fluctuations, over the course of the 200 ns simulation. Compound 6 after initial deviations, indicated stable pose throughout the simulation. Conversely, compounds 5 and 7 exhibited a lack of stability, failing to achieve a stable conformation. Hydrogen bond analysis revealed varying degrees of interaction between the ligands and the receptor. Notably, compounds 1, 3, 4, 8, and 10 established multiple hydrogen bonds with the receptor, while compound 6 exhibited a remarkable increase in hydrogen bond formation over time, peaking at eight bonds during the simulation. However, compounds 2 and 9 showed infrequent hydrogen bond formation. Per residue decomposition analysis of the residues in the MD trajectory indicated residues namely His137, Glu172, Arg183, Arg2258 contributing majorly in non-covalent interaction with the protein in most of the ligands (Fig 9A–9E). Overlapping of the protein-ligand conformations from time frame 50ns, 100ns, 150ns and 200ns also indicated the ligands 1, 3, 4, 6 and 10 indicated stable poses with low deviations in the binding pocket (Fig 10A–10B). For compounds namely 2, 5, 7, 8 and 9, the overlapped conformations of the ligands were highly divergent indicating low stability. Although, we highlighted the presence of a single prominent global minimum in the free energy landscape (FEL) plots for compounds 1, 3, 4, 6, and 10, as depicted in Fig 11. While the identification of a global minimum suggests stability, it is not the sole criterion for assessing complex stability and binding affinity. Factors such as the free energy level of the most stable state, conformational entropy, and conformational diversity also play crucial roles in determining binding affinity. We also carried out ADMET studies of top selected molecules using ADMETlab webserver.

The results have been summarized in Table 5. Based on a meticulous examination of the RMSD plots, in conjunction with an assessment of the hydrogen bonding pattern and a water-based scoring scheme, it is possible to deduce that five specific molecules, namely 1, 3, 4, 6, and 10, exhibit characteristics that warrant prioritization for subsequent evaluation.

**Table 5:** The table summarize the various variables which are used for calculating ΔG value. GMX MMPBSA score was calculated using last 50 ns trajectory of the MD simulation file.

MOLECULE	ΔVDWAALS	ΔEEL	ΔEGB	ΔESURF	ΔGGAS	ΔGSOLV	ΔTOTAL
**1**	-65.71	-46.92	59.63	-10.30	-112.63	49.34	-63.29
**2**	-44.26	-26.04	37.07	-7.04	-70.29	30.04	-40.26
**3**	-55.26	-21.45	34.37	-8.84	-57.67	29.07	-47.60
**4**	-53.93	-55.64	57.82	-8.12	-109.58	49.70	-59.87
**5**	-56.76	-19.40	40.30	-8.31	-76.16	31.99	-44.17
**6**	-54.69	-71.14	80.23	-9.18	-125.83	71.05	-54.77
**7**	-50.97	-17.73	38.13	-7.66	-68.70	30.47	-38.24
**8**	-41.37	-6.33	25.58	-5.41	-47.70	20.17	-27.53
**9**	-47.59	-6.12	31.13	-6.21	-53.70	24.92	-28.78
**10**	-62.65	-20.85	47.13	-8.79	-83.50	38.34	-45.16
**Control**	-53.54	-23.06	41.58	-7.43	-76.60	34.15	-42.45

## 4. Conclusion

This paper introduces a robust computational model for the purpose of rationalizing the design of GPR40 protein agonists. By utilizing a substantial collection of protein-ligand crystal structures, we successfully elucidated the distinctive features of the protein’s allosteric binding site. This region encompasses a cavity that assumes a pivotal function in the binding of agonists. Our computational structural analysis identified critical residues, such as Arg183, Tyr402, and Phe87, which are crucial for the stability and binding affinity of agonists within the GPR40 binding site. Hydrogen bond interactions with Arg2240 were also noted, which were not previously documented in literature, indicating new potential targets for enhancing binding affinity. The developed pharmacophore model consists of five features: three aromatic features (R1, R2, and R3), one hydrophobic feature (Hy1), and one acceptor feature (A1). By employing a screening methodology that utilizes a structural ligand-based pharmacophore, we successfully eliminated a mere 0.01867% of hit compounds from an extensive library. Furthermore, we successfully identified ten compounds that have a higher docking score compared to TAK-875. Glide Docking Scores provide a quick assessment of binding affinity by evaluating the fit within the binding site, whereas MMPBSA Scores offer a detailed evaluation of binding free energy, accounting for solvation effects and entropic contributions. Furthermore, structural modifications in agonist design may increase potency for molecules, especially, by incorporating a carboxylic group to establish critical interactions with Arg183 and Tyr2240. Positioning aromatic groups to enhance pi-pi interactions with residues like Tyr91 and Phe87, and ensuring a hydrophobic pocket near the hydrophobic goove of GPR40 to enhance binding. The stability of the top five molecules (1, 3, 4, 6, and 10) was seen in their interactions with the receptor in the binding groove during the MD studies, suggesting strong solvation based binding affinity compared to the TAK-875. All of the aforementioned compounds exhibited a single prominent global minimum in the free energy landscape plot, suggesting that they may have attained a stable conformation with the receptor. This suggests that their strong affinity to the receptor can be attributed to their increased interactions with the receptor through hydrogen bonding and other non-covalent interactions. The compounds shortlisted through the approach can be further analyzed in order to assess their potential therapeutic effects on the GPR40 protein. Validation of these compounds will be conducted in our next investigations through the utilization of a cell line-based evaluation. Once validated, these compounds have the potential to be utilized as primary candidates for optimization studies or subsequent in vivo investigations. Regarding the future of this work, we plan to broaden our scope to include other drug targets related to metabolic and neurological diseases. The computational methodology developed in our research can be adapted to facilitate the discovery of previously unidentified agonist compounds for various targets beyond GPR40. This approach may be applied to various molecular databases, thereby enhancing the likelihood of identifying novel molecules with therapeutic potential for targeting GPR40. However, this study has limitations. The computational predictions need to be validated experimentally to confirm the biological activity of the identified compounds. Additionally, our model relies on the accuracy of available crystal structures, and the dynamic nature of protein-ligand interactions might not be fully captured. Future work will involve integrating more dynamic simulations and experimental validation to address these limitations. By refining our model and incorporating more comprehensive datasets, we aim to improve the predictive power of our computational approach, thereby advancing the field of drug discovery.
